# Distinct Basal Metabolism in Three Mouse Models of Neurodevelopmental Disorders

**DOI:** 10.1523/ENEURO.0292-20.2021

**Published:** 2021-04-15

**Authors:** Caitlin Menzies, Shama Naz, David Patten, Thierry Alquier, Brian M. Bennett, Baptiste Lacoste

**Affiliations:** 1 Neuroscience Program, Ottawa Hospital Research Institute, Ottawa, Ontario K1H 8L6, Canada; 2 Faculty of Medicine, Department of Cellular and Molecular Medicine, University of Ottawa, Ottawa, Ontario K1H 8M5, Canada; 3 University of Ottawa Metabolomics Core Facility, Faculty of Medicine, Ottawa, Ontario K1H 8M5, Canada; 4 Montreal Diabetes Research Center and Centre de Recherche du Centre Hospitalier de l'Université de Montréal (CRCHUM), Montréal, Quebec H1W 4A4, Canada; 5 Department of Medicine, Université de Montréal, Montréal, Quebec H3T 1J4, Canada; 6 Faculty of Health Sciences, Department of Biomedical and Molecular Sciences and Centre for Neuroscience Studies, Queens University, Kingston, Ontario K7L 3N6, Canada; 7 University of Ottawa Brain and Mind Research Institute, Ottawa, Ontario K1H 8M5, Canada

**Keywords:** 16p11.2 deletion syndrome, basal metabolism, Down syndrome, fragile X syndrome, neurodevelopmental disorders, plasma metabolites

## Abstract

Prevalence of metabolic disturbances is higher among individuals with neurodevelopmental disorders (NDDs), yet this association has been largely overlooked. Investigation into human disease remains challenging, as a complete pathophysiological understanding relies on accurate modeling and highly controlled variables. Genetically engineered mouse models are widely used to gain insight into the biology of human NDDs, but research focus has been on behavioral and neurophysiological abnormalities. Such models not only allow for evaluating usefulness in reproducing human features, including similarities and discrepancies with rodent phenotypes, but they also represent a unique opportunity to observe and quantify novel anomalies. Here, we present the first characterization and comparison of basal metabolism in three mouse models of NDDs, namely, Down syndrome (DS; *Dp(16)Yey/+* mice), 16p11.2 deletion syndrome (16pDel; *16p11.2^df/+^* mice), and fragile X syndrome [FXS; *Fmr1* knock-out (KO) mice] and their wild-type (WT) counterparts. Using the Comprehensive Lab Animal Monitoring System (CLAMS) coupled to EchoMRI, as well as quantification of key plasma metabolites by liquid chromatography mass spectrometry (LC-MS), our *in vivo* study reveals that each mouse model expresses a unique metabolic signature that is sex-specific, independent of the amount of food consumed and minimally influenced by physical activity. In particular, we identify striking differences in body composition, respiratory exchange ratio (RER), caloric expenditure (CE), and concentrations of circulating plasma metabolites related to mitochondrial function. Providing novel insight into NDD-associated metabolic alterations is an essential prerequisite for future preclinical and clinical interventions.

## Significance Statement

Most studies on neurodevelopmental and autism spectrum disorders (ASDs) have focused on neurophysiological mechanisms, yet these disorders are also associated with metabolic abnormalities in humans. Despite this known association, the link between metabolic imbalance and neurodevelopmental disorders (NDDs) has been largely overlooked, particularly in a fundamental research setting. Here, to address this knowledge gap, we performed the first systematic characterization of basal metabolism in mouse models of Down syndrome (DS), 16p11.2 deletion syndrome (16pDel), and fragile X syndrome (FXS), revealing unique metabolic signatures. This work provides a basis for future studies aimed at understanding mechanisms underlying metabolic dysfunction in NDDs.

## Introduction

Down syndrome (DS), 16p11.2 deletion syndrome (16pDel), and fragile X syndrome (FXS) are neurodevelopmental disorders (NDDs) of distinct genetic origins commonly associated with intellectual disability, developmental delay and autism spectrum disorders (ASDs; Miller et al., 2009; Hanson et al., 2010; McLennan et al., 2011; Shields et al., 2017). These particular NDDs are also associated with higher incidence of obesity, hypertension, hormonal dysfunction, heart defects and diabetes in comparison to the general population (McLennan et al., 2011; Miller et al., 2009; Croen et al., 2015; Shields et al., 2017; Flygare Wallén et al., 2018).

DS is caused by the presence of a third copy of chromosome 21. DS individuals display developmental delay and behavioral abnormalities (Mazurek and Wyka, 2015), and infants are often premature with low birthweights and heart defects leading to higher rates of neonate mortality (Vis et al., 2009; Mazurek and Wyka, 2015). Adult DS patients display obesity, insulin resistance, type 2 diabetes and decreased cardiovascular fitness (Vis et al., 2009; Mendonca et al., 2010; Real de Asua et al., 2014; Bertapelli et al., 2016; Shields et al., 2017). Despite experiencing multiple cardiovascular risk factors, DS individuals display lower rates of coronary artery disease and atherosclerotic damage, particularly among males (Vianello et al., 2013; Sobey et al., 2015). 16pDel originates from a hemizygous deletion at the 16p11.2 locus, resulting in the loss of ∼500 kb of DNA and haploinsufficiency of ∼30 genes. Individuals harboring a 16p11.2 deletion are characterized by developmental delay and speech and language problems (Miller et al., 2009; Hanson et al., 2010; Horev et al., 2011). 16pDel is also associated with obesity and hyperinsulinaemic hypoglycemia (Kostopoulou et al., 2019). Male carriers are at higher risk of developing hypertension, whereas female carriers are at greater risk of developing type 2 diabetes (Croen et al., 2015; Flygare Wallén et al., 2018). Finally, FXS is caused by an expansion of CGG repeats in the promoter region of the *FMR1* gene, which prevents production of fragile X mental retardation protein (FMRP; Hoogeveen and Oostra, 1997; McLennan et al., 2011; Dahlhaus, 2018). FXS is one of the most common genetic causes of moderate-to-severe intellectual impairments but is also associated with cardiovascular disease, obesity and hypertension (Crabbe et al., 1993; McLennan et al., 2011). As FXS is an X-linked syndrome, males are more commonly affected by FXS and typically display severe phenotypes. Females may harbor a heterozygous or homozygous FXS mutation (Linden et al., 1999; Bartholomay et al., 2019). Heterozygous females produce ∼80% of the FMRP levels and display mild phenotypes (Bartholomay et al., 2019). Rarer homozygous FXS females display phenotypes similar to male FXS individuals (Linden et al., 1999; Bartholomay et al., 2019).

Genetically engineered mouse models of DS, 16pDel, and FXS have proven reliable tools in preclinical research (Bakker et al., 1994; Roubertoux and Carlier, 2010; Yang et al., 2015; Arbogast et al., 2016; Herault et al., 2017; Lovelace et al., 2018). To model DS in this study, we used *Dp(16)Yey/+* mice that harbor trisomy of the orthologous genes of human chromosome 21 (Hsa21) that are present on Mmu16. These mice embody cognitive and developmental aspects reminiscent of human DS (Li et al., 2007; Roubertoux and Carlier, 2010; Yu et al., 2010; Herault et al., 2017). Heterozygous *16p11.2^df/+^* mice represent a robust 16pDel model. They possess a deletion (∼440 kb, 27 genes) of the 7qF3 region of synteny conservation with the human 16p11.2 locus (Horev et al., 2011). Compared with their wild-type (WT) littermates, *16p11.2^df/+^* mice display alterations including social interactions deficits and hyperactivity. These mice also display lower birthweight and decreased adiposity in adults, improved glucose tolerance, decreased leptin and free fatty acid levels, as well as cerebrovascular deficits (Horev et al., 2011; Portmann et al., 2014; Arbogast et al., 2016; Ouellette et al., 2020). Finally, *Fmr1*^−/−^ knock-out (KO) mice are commonly used to model human FXS. *Fmr1*^−/−^ mice harbor a KO allele of the *Fmr1* gene located on the X chromosome. Although different from human FXS, *Fmr1*^−/−^ mice lack FMRP expression (Hoogeveen and Oostra, 1997; Dahlhaus, 2018). *Fmr1*^−/−^ mice display phenotypes including enhanced sensitivity to sensory stimuli, hyperactivity and physical characteristics (Hoogeveen and Oostra, 1997; Dahlhaus, 2018; Lovelace et al., 2018). These mice also display increased glucose tolerance and insulin sensitivity, as well as a shift toward lipid utilization (Leboucher et al., 2019).

Despite this context, a systematic characterization of basal metabolism in these three mouse models is lacking. Here, we performed a comprehensive assessment of basal metabolism of DS, 16pDel, and FXS mice by measuring body composition, physiological indices and selected plasma metabolites.

## Materials and Methods

All animal procedures were conducted in accordance to guidelines of the Canadian Council on Animal Care.

### Animals

*Dp(16)Yey/+* “DS” mice (stock #013530; mixed B6/129 background) were obtained from The Jackson Laboratory through the Cytogenetics and Down Syndrome Resource funded by a contract from the National Institute of Child Health and Human Development/National Institutes of Health (275201000006C-3-0-1). These mice harbor trisomy of the Mmu16 region of synteny with the Down Syndrome Critical Region (DSCR) on Hsa21 and carry 113 genes orthologous to genes on Hsa21 (Roubertoux and Carlier, 2010; Herault et al., 2017). *16p11.2^df/+^* “16pDel” mice (Horev et al., 2011) were purchased from The Jackson Laboratory (stock #013128; mixed B6/129 background). Heterozygous *16p11.2^df/+^* mice possess a deletion of the 7qF3 region of synteny conservation with the human 16p11.2 locus. *Fmr1*^−/−^ “FXS” mice (Bakker et al., 1994) were purchased from The Jackson Laboratory (stock #004624, mixed FVB/129 background). These mice lack of expression of the *Fmr1* gene product.

### Mouse husbandry

All mice were bred in-house and housed maximum five per cage at room temperature with free access to water and food. Animals were maintained on Teklad Global 18% Protein Rodent Diet (Harlan Laboratories, Teklad Diets) composed in part of 18.6% protein, 6.2% fat, 3.5% fiber, and 44.2% carbohydrates. Male *16p11.2^df/+^* were crossed with WT females to obtain hemizygous *16p11.2^df/+^* mice and WT littermates. *Fmr1*^−/−^ males were bred with *Fmr1*^−/−^ females to obtain *Fmr1*^−/−^ progeny, and *Fmr1*^−/−^ males were bred with FVB females to obtain *Fmr1*^+/−^ heterozygous animals. Male *Dp(16)Yey/+* mice were bred with female C57BL/6J mice (The Jackson Laboratory) to obtain hemizygous *Dp(16)Yey/+* mice and WT littermates.

### Genotyping

*16p11.2^df/+^* mice and WT littermates were genotyped by PCR using the two following primers: 5′-CCTCATGGACTAATTATGGAC-3′ (forward) and 5′-CCAGTTTCACTAATGACACA-3′ (reverse). *Fmr1*^−/−^ mice and WT littermates were genotyped by PCR using the three following primers: 5′-CACGAGACTAGTGAGACGTG-3′ (mutant forward), 5′-TGTGATAGAATATGCAGCATGTGA-3′ (WT forward), and 5′-CTTCTGGCACCTCCAGCTT-3′ (common). *Dp(16)Yey/+* mice and WT littermates were genotyped by PCR by a commercial vendor (Transnetyx) using proprietary primers.

#### *In vivo* basal metabolism assessment

Live, awake 10-week-old mice were weighed, placed in an enclosed tube and inserted into the Echo-MRI machine (EchoMRI-700, EchoMRI LLC) for 2–3 min. EchoMRI device employs magnetic resonance imaging to quantify total body weight, lean mass and fat mass in grams. Immediately following EchoMRI scanning, mice were individually housed at thermo-neutrality (28°C) into Comprehensive Lab Animal Monitoring System (CLAMS) cages (CLAMS-CF and Oxymax software, Columbus Instruments) for metabolic measurements using indirect calorimetry (IC). A thermo-neutral environment eliminates the need for the body to expend energy heating the body (Ramos-Jimenez et al., 2008; Gupta et al., 2017; Mtaweh et al., 2018). CLAMS cages are open circuit systems in which subjects breathe in gases that match atmospheric composition in an airtight acrylic chamber meant to mimic a home-cage, thermo-neutral environment with open access to food and water. CLAMS chambers are of the following dimensions. Livable area: 7 inches (17.75 cm) in diameter, 5.625 inches (14.25 cm) in ceiling height. Overall size with base and stand: 15 inches (38 cm) wide, 11 inches (28 cm) deep, 23 inches (58.4 cm) high. Animals were monitored daily for well-being. Equipment was calibrated before data collection by the University of Ottawa’s Behavioural Core services and checked daily to ensure proper functioning. The CLAMS software automatically calculates output measures of volume of O_2_ consumption (VO_2_), volume of CO_2_ production (VCO_2_), respiratory exchange ratio (RER), caloric expenditure (CE), and food intake, as well as physical activity levels. The physiological effects of stress are minimized by allowing 24 h of habituation before data collection and minimal noise in the experimental area (Arch, et al., 2006; Ramos-Jimenez et al., 2008; Gupta et al., 2017).

IC is based on the principal that the conversion of carbohydrates, fats and proteins into chemical energy results in a substance-specific ratio of O_2_ required for catabolism of macronutrients and CO_2_ produced as a by-product (Ramos-Jimenez et al., 2008; Watson et al., 2014; Arch et al., 2006; Mtaweh et al., 2018). A number of factors must be considered for accurate IC measurement. Total CE measured by CLAMS is a composite of resting energy expenditure (REE), activity-related energy expenditure (AEE), and the thermic effect of feeding (TEF). REE is the energy required for the body to conduct the biochemical reactions required to sustain life and accounts for most bodily energy expenditure of mammals. AEE refers to energy used while performing elective movement, such as walking or running. TEF refers to the energy lost in the form of heat in the process of substrate utilization (Mtaweh et al., 2018). CLAMS cages provide an environment that minimizes the contribution of AEE and TEF. The CLAMS chambers where mice are housed are small and limit movement of the animal thus limiting AEE. TEF is mitigated by continuous feeding throughout the experiment and housing animals in a thermo-neutral environment (Ramos-Jimenez et al., 2008; Mtaweh et al., 2018).

Gas exchanges were measured every 26 min over a 72-h period consisting of an initial 24-h habituation period followed by 48 h of data collection. The RER value represents the ratio of VCO_2_ produced to VO_2_ consumed. Total CE was calculated by a multi-step equation where a “calorific value,” a standardized number of kilocalories generated per liter of O_2_ consumed, was first obtained by using RER. This calorific value was then multiplied by the VO_2_ of the organism to obtain the number of kilocalories used per unit of time. Food intake was measured as the cumulative amount of food consumed from the beginning of the experiment at each measurement period. Physical activity of animals was measured via an infrared beam system. Data are presented in terms of “beam breaks” where the number of times different beams were interrupted by the animal during the interval of time from one sampling session to the next.

CLAMS data were expressed both in absolute terms, and normalized to body weight and to lean mass, as recommended for comparison of populations with differential anthropomorphic characteristics or metabolic health status (Arch et al., 2006; Watson et al., 2014; Van Eersel et al., 2017; Mtaweh et al., 2018). Moreover, it is suggested that RER is calculated from absolute values of O_2_ and CO_2_ exchanges (Arch et al., 2006; Tschöp et al., 2011). Normalization of CE to lean mass facilitates comparison between groups of different body composition (Mtaweh, et al., 2018).

#### Targeted plasma metabolome profiling

Plasma levels of selected metabolites were quantified by liquid chromatography mass spectrometry (LC-MS). Sample temperature was maintained on ice or dry ice where possible, and all solvents were MS grade and pre-equilibrated to −20°C.

##### Metabolite extraction

Mouse saphenous vein blood (200 μl) was collected in prechilled EDTA tubes, and centrifuged for 10 min at 4500 rpm at 4°C. Metabolites from 50 μl of the resulting plasma were extracted with 600 μl of a 1:1:1 mixture of methanol:acetonitrile:water. Samples were vortexed and 600 μl of dichloromethane plus an additional 300-μl water were added for liquid-liquid extraction. Samples were vortexed, incubated on ice for 10 min and then centrifuged for 10 min at 4000 rpm at 4°C. The resulting upper phase, consisting of polar metabolites, was separated into two fractions (for +ESI and –ESI injections), dried with a refrigerated (−4°C) centrivap concentrator (Labconco) and stored at −80°C before LC-MS analyses.

##### LC-MS metabolite quantification

Samples were resuspended with 75% acetonitrile, cleared by centrifugation and run on an Agilent 6545B Q-TOF mass spectrometer equipped with a 1290 Infinity II ultra-high performance LC (Agilent Technologies). Continuous internal mass calibration was executed using signals from purine [12,000 full width at half maximum (FWHM) resolution] and hexakis (1H, 1H, 3H-tetrafluoropropoxy) phosphazine (24,000 FWHM resolution). All study samples were randomized before analysis and run using both high and low pH hydrophilic interaction chromatography (HILIC-Z) in negative and positive ionization polarities, respectively. HILIC separation was obtained using the Poroshell 120 HILIC-Z column (2.1 × 100 mm, 2.7 μm; Agilent) and corresponding guard column. For negative ion mode chromatography, mobile phase A consisted of water and mobile phase B acetonitrile/water (85/15, v/v), both with 10 mM ammonium acetate and 5 μm medronic acid following the gradient: 0–2 min 96% B; 2–12 min linear gradient to 60% B; 12–15 min linear gradient to 60% B, 15–16 min back to initial conditions and re-equilibrated in 96% B for 8 min. For positive ion mode chromatography, mobile phase A consisted of water and mobile phase B acetonitrile/water (90/10, v/v), both with 10 mm ammonium formate and 0.1% formic acid following the gradient: 0–1 min 98% B; 1–1.5 min linear gradient 90% B, 1.5–5 min linear gradient 80% B, 5–8 min linear gradient 60% B, 8–10.7 linear gradient 5% B, 10.7–12 min 98% B, 12–12.7 min linear gradient to initial conditions and re-equilibrated for 3.6 min. Autosampler and column temperature were maintained at 4°C and 30°C, respectively. Samples were analyzed using 0.25 ml/min flow rate with an injection volume of 2 μl. MS detection settings were as follows: N_2_ drying gas temperature 200°C (–ESI) and 200°C (+ESI); N_2_ drying gas flow 10 l/min; sheath gas temperature 300°C (–ESI) and 225°C (+ESI); sheath gas flow 12 l/min; nebulizer pressure 40 psig, capillary voltage 3000 V, nozzle voltage 0 V and fragmentor voltage of 125 V. MS data were collected for m/z range of 60–1050 at the acquisition rate of two spectra/s in the extended dynamic range mode (2 GHz).

##### Metabolite identification and analysis

Metabolite identification was confirmed by exact mass, retention time and subsequent MS/MS fragmentation of metabolite standards and quality control samples. These identifications correspond to Metabolomics Standards Initiative identification level 1 (Sumner et al., 2007). A targeted list of metabolites was quantified (relative quantification) by external standard calibration curves with Mass Hunter Quant (Agilent).

### Statistics

No statistical methods were used to predetermine sample size. Sample size (*n* = 10 per sex/genotype) was similar to previous reports (Horev et al., 2011; Portmann et al., 2014; Arbogast et al., 2016). Researchers were blind to genotype throughout data collection and analysis. In whisker box plots, boxes represent interquartile range (IQR), the median value is represented by a line through the box, and whiskers represent maximum and minimum values. In linear graphs, the line represents the mean and shadow represents SEM. Group differences were analyzed by two-way ANOVA and a Sidak’s multiple comparisons *post hoc* test. Statistical significance, depicted by asterisks in plots, was considered when *p* < 0.05. All statistical tests were performed using GraphPad Prism 9.0 Software.

## Results

### Body composition of DS, 16pDel, and FXS mice measured by EchoMRI

**Figure 1.**
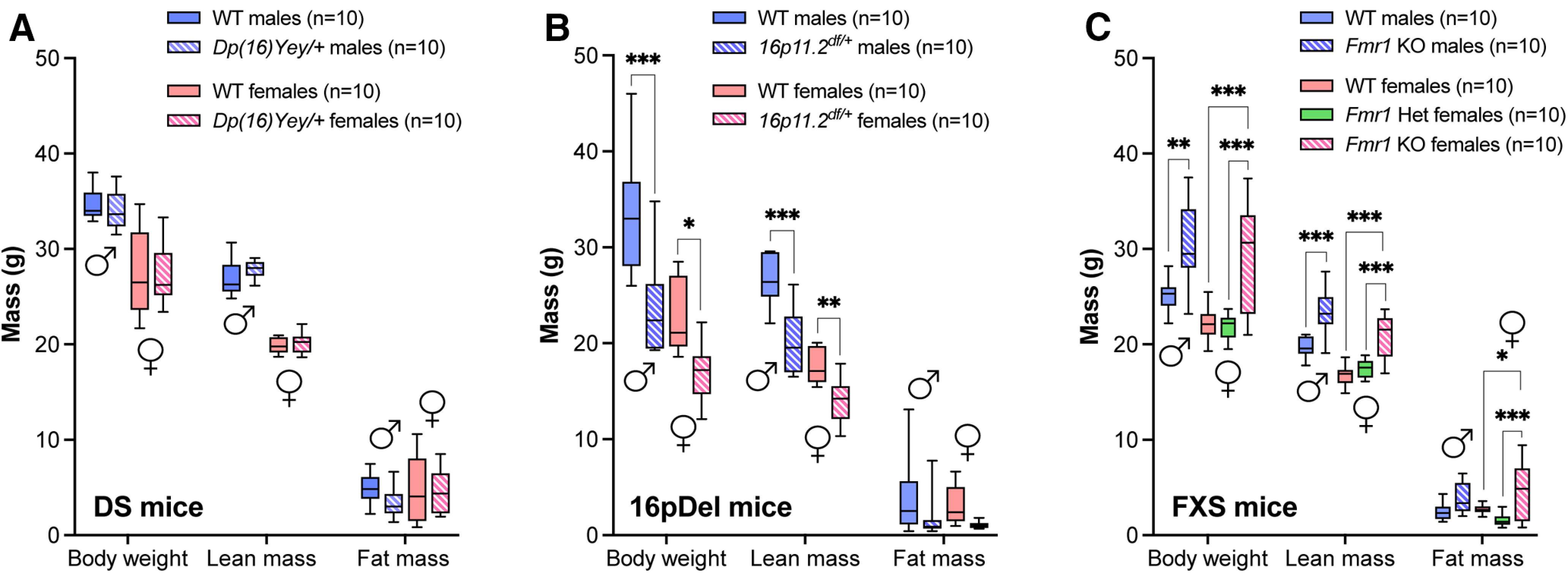
Analysis of body weight, lean mass, and fat mass in DS, 16pDel, and FXS mice by EchoMRI. ***A***, Weight, lean mass, and fat mass (g) of WT and *Dp(16)Yey/+* male and female mice. ***B***, Weight, lean mass, and fat mass (g) of WT and *16p11.2^df/+^* male and female mice. ***C***, Weight, lean mass, and fat mass (g) of WT and *Fmr1* KO (homo/heterozygous) male and female mice. WT, wild type; DS, Down syndrome; 16pDel, 16p11.2 deletion syndrome; FXS, fragile X syndrome. All data are whisker boxes (min to max, center line indicating median; *n* = 10 animals per group). Asterisks depict significant differences between groups; *p <* *0.05, **p <* *0.01, ***p <* *0.001 (two-way ANOVA and Sidak’s *post hoc* test). ♂: males; ♀: females.

In humans, DS, 16pDel, and FXS are all associated with alterations in body composition, including altered adipose tissue content and body size (Vis et al;, 2009; Horev et al., 2011; McLennan et al., 2011; Real de Asua et al., 2014; Gimeno-Ferrer et al., 2018). We first sought to examine body composition of the corresponding mouse models using EchoMRI to acquire quantitative measures of weight, lean mass and fat mass. No significant difference in weight, lean or fat mass was measured between *Dp(16)Yey/+* mice and their WT littermates (Fig. 1*A*), but a main effect of sex was detected for body weight (*F*_(1,18)_ = 63.09; *p* < 0.0001) and lean mass (*F*_(1,18)_ = 271.6; *p* < 0.0001). We found that *16p11.2^df/+^* mice weighed significantly less and had significantly lower proportions of lean mass compared with their WT littermates (Fig. 1*B*). The *Fmr1*^−/−^ mice, however, weighed more and had higher lean and fat mass content when compared with their WT counterparts (Fig. 1*C*). As the mutation in *Fmr1* underlying FXS is X-linked, females may be either homozygous or heterozygous for the mutation (Linden et al., 1999). We therefore included heterozygous *Fmr1*^+/−^ females in our study. There was no difference in weight, lean or fat mass between female *Fmr1*^+/−^ and WT mice (Fig. 1*C*). These results demonstrate that genetic variations associated with DS, 16pDel, and FXS in mice affect body weight, lean mass, and fat mass in a distinct manner.

### Basal metabolism indices measured by CLAMS in DS, 16pDel, and FXS mice

Physiologic measures for each mouse model were then collected over a 48-h period (i.e., two day/night cycles) by housing mice in the CLAMS. Data were averaged over a 24-h cycle. CLAMS cages allow for a tight control of environmental factors, such as temperature, that could confound metabolic calculations. Body composition data obtained from the EchoMRI (body weight or lean mass) were used to normalize CLAMS-generated metabolic data (see Materials and Methods).

**Figure 2.**
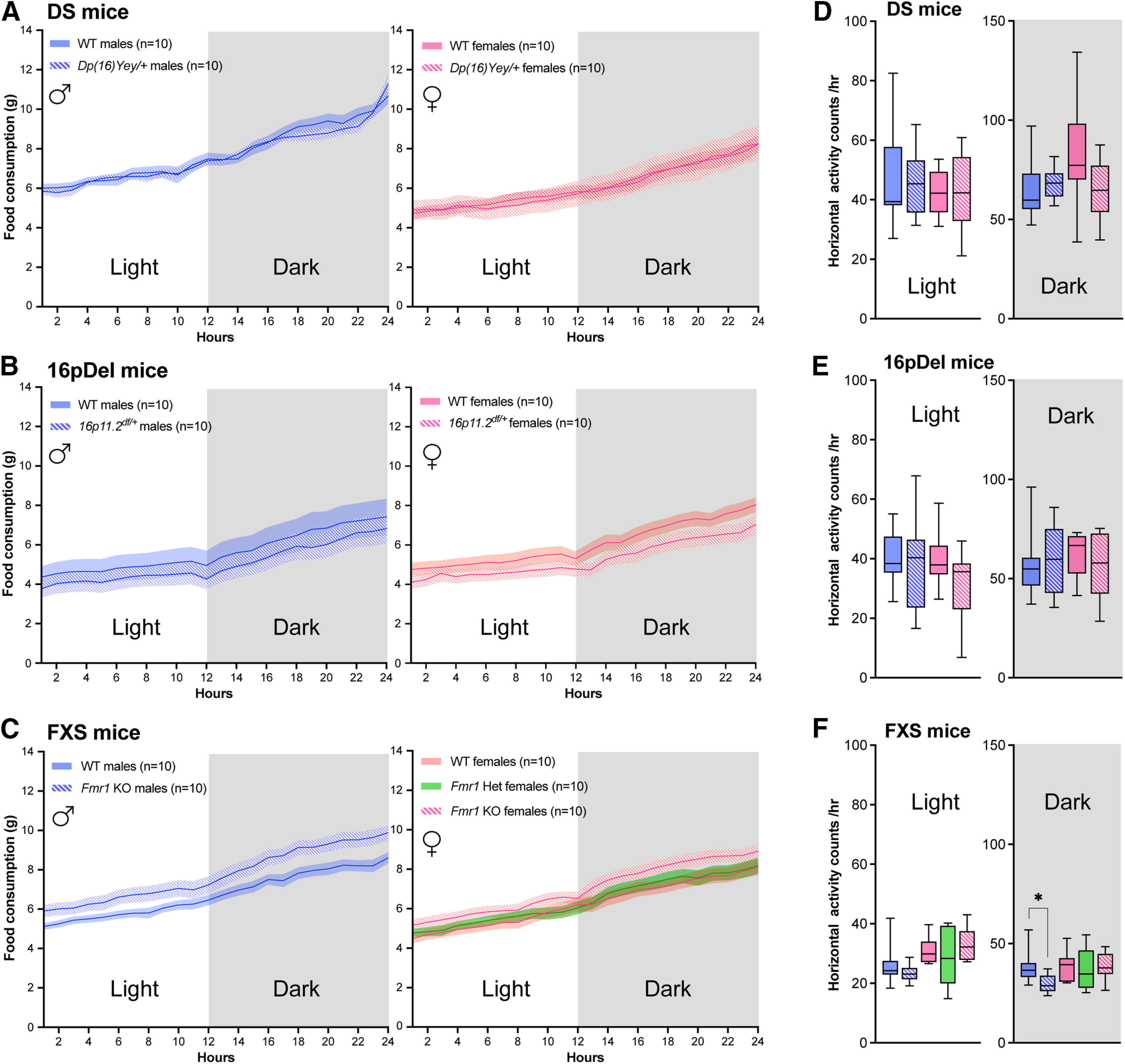
Cumulative food consumption and activity levels of DS, 16pDel, and FXS mice measured in CLAMS. ***A–C***, Average cumulative food consumption in grams shown over a 24-h cycle for WT and *Dp(16)Yey/+* male and female mice (***A***); WT and *16p11.2^df/+^* male and female mice (***B***); WT and *Fmr1* KO (homo/heterozygous) male and female mice (***C***). ***D–F***, Activity levels (horizontal beam breaks) in CLAMS cages for WT and *Dp(16)Yey/+* male and female mice (***D***); WT and *16p11.2^df/+^* male and female mice (***E***); WT and *Fmr1* KO (homo/heterozygous) male and female mice (***F***). The night phase of testing is depicted by gray shaded area. WT, wild type; DS, Down syndrome; 16pDel, 16p11.2 deletion syndrome; FXS, fragile X syndrome. Data are whisker boxes (min to max, center line indicating median) in ***A****–****C***, or mean ± SEM in ***D–F*** (*n* = 10 animals per group); *p <* *0.05 (two-way ANOVA and Sidak’s *post hoc* test). ♂: males; ♀: females.

Body composition and metabolic variables are highly influenced by food intake and engagement in physical activity (qualitatively and quantitatively; Vis et al;, 2009; Mazurek and Wyka, 2015; Bertapelli et al., 2016; Peretti et al., 2019). To account for the influence of food intake on body composition and subsequent metabolic indices, all mice were fed the same chow diet and the cumulative amount of food consumed by each animal was measured over the duration of the experiment. No significant difference in food consumption was found in all three mouse models (Fig. 2*A–C*). Physical activity (i.e., number of horizontal laser beam breaks) of each animal was also recorded in CLAMS cages. Activity levels were largely similar between mutant and WT mice from all models (Fig. 2*D–F*). We only detected a main effect of sex for FXS mice (day: *F*_(1,18)_ = 17.48; *p* < 0.001; night: *F*_(1,18)_ = 4.835; *p* < 0.05) and an interaction between sex and genotype for DS mice at night (*F*_(1,18)_ = 5.758; *p* < 0.05) and a slight, albeit significant, difference was found between *Fmr1*^−/−^ males and their WT counterparts at night (Fig. 2*F*). Hence, these data suggest that differences identified in our study are not because of feeding behaviors and are minimally influenced by activity levels. We thus hypothesized that metabolic differences observed hereafter result from changes in basal energy metabolism.

**Figure 3.**
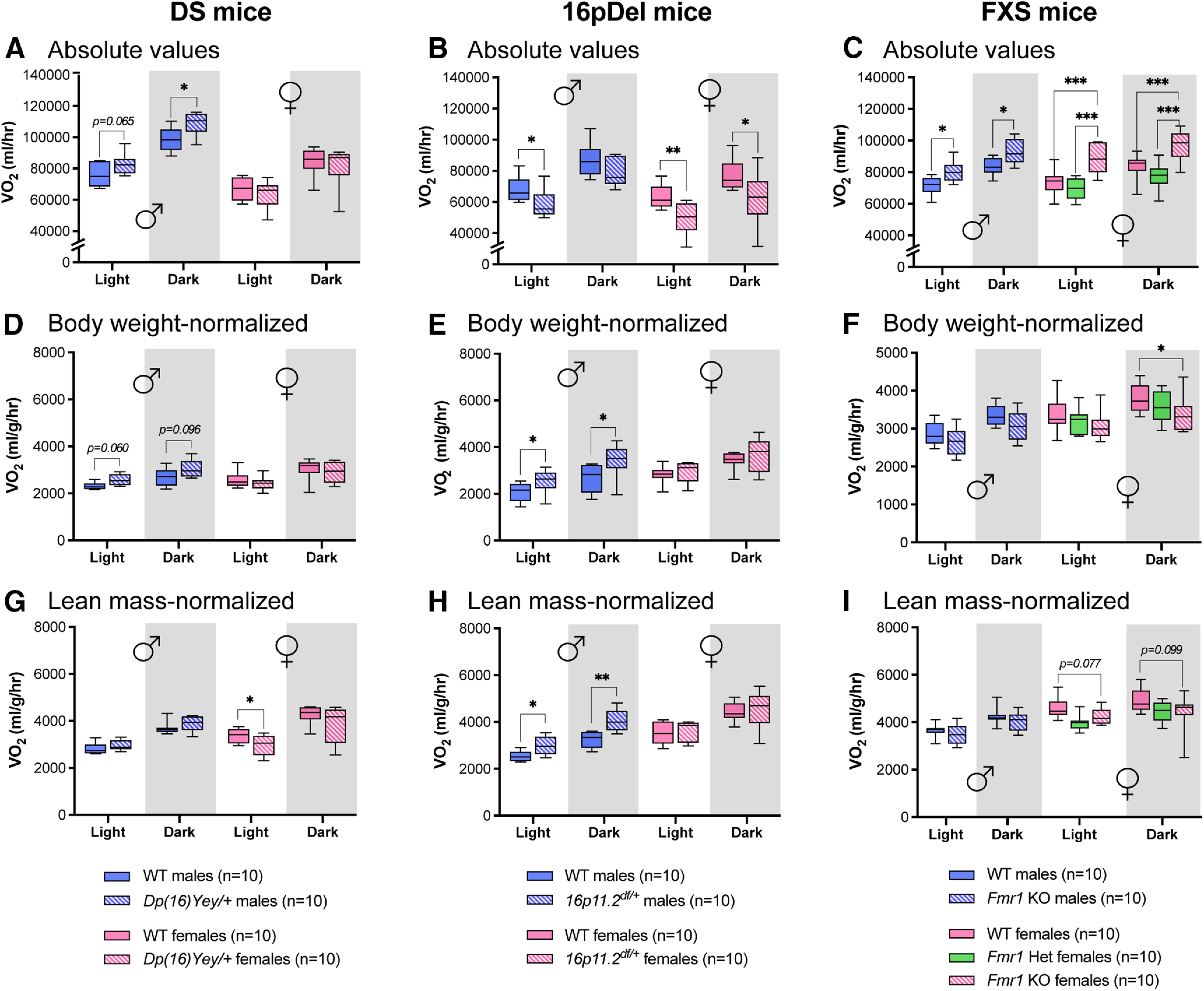
VO_2_ by DS, 16pDel, and FXS mice measured in CLAMS. ***A–C***, Absolute values of average VO_2_ during day (light) and night (dark) in WT and *Dp(16)Yey/+* male and female mice (***A***); WT and *16p11.2^df/+^* male and female mice (***B***); WT and *Fmr1* KO (homo/heterozygous) male and female mice (***C***). ***D–F***, Average VO_2_ during day and night normalized to body weight in WT and *Dp(16)Yey/+* male and female mice (***D***); WT and *16p11.2^df/+^* male and female mice (***E***); WT and *Fmr1* KO (homo/heterozygous) male and female mice (***F***)**. *G****–****I*,** Average VO_2_ during day and night normalized to lean mass in WT and *Dp(16)Yey/+* male and female mice (***G***); WT and *16p11.2^df/+^* male and female mice (***H***); WT and *Fmr1* KO (homo/heterozygous) male and female mice (***I***). The night phase of testing is depicted by gray shaded areas. WT, wild type; DS, Down syndrome; 16pDel, 16p11.2 deletion syndrome; FXS, fragile X syndrome. Data are whisker boxes (min to max, center line indicating median); *n* = 10 animals per group. Asterisks depict significant differences between groups; *p <* *0.05, **p <* *0.01, ***p <* *0.001 (two-way ANOVA and Sidak’s *post hoc* test); *p* value is indicated when approaching significance. ♂: males; ♀: females.

When collecting and analyzing CLAMS data on VO_2_, we found that *Dp(16)Yey/+* males consumed more O_2_ than females and WT males at night (Fig. 3*A*). No difference was detected among body weight-normalized values (Fig. 3*D*); however, when normalized to lean mass, *Dp(16)Yey/+* females appeared to consume less O_2_ during the day compared with sex-matched WT littermates (Fig. 3*G*).

*16p11.2^df/+^* mice consumed less O_2_ than their WT counterparts, a difference more pronounced in females (Fig. 3*B*). Interestingly, when normalized to body weight or lean mass, a male-specific increase in O_2_ consumption was observed among *16p11.2^df/+^* mice (Fig. 3*E*,*H*). Conversely, *Fmr1*^−/−^ mice consumed more O_2_ than their sex-matched WT littermates (Fig. 3*C*). When normalized to body weight or lean mass, *Fmr1*^−/−^ females appeared to consume less O_2_ than their WT counterparts (Fig. 3*F*,*I*). Similar VO_2_ was found between *Fmr1*^+/−^ and WT females (Fig. 3*C*,*F*,*I*). These results demonstrate that rates of O_2_ consumption are inconsistent between the DS, 16pDel, and FXS mouse models, and that these phenotypes are sex specific.

**Figure 4.**
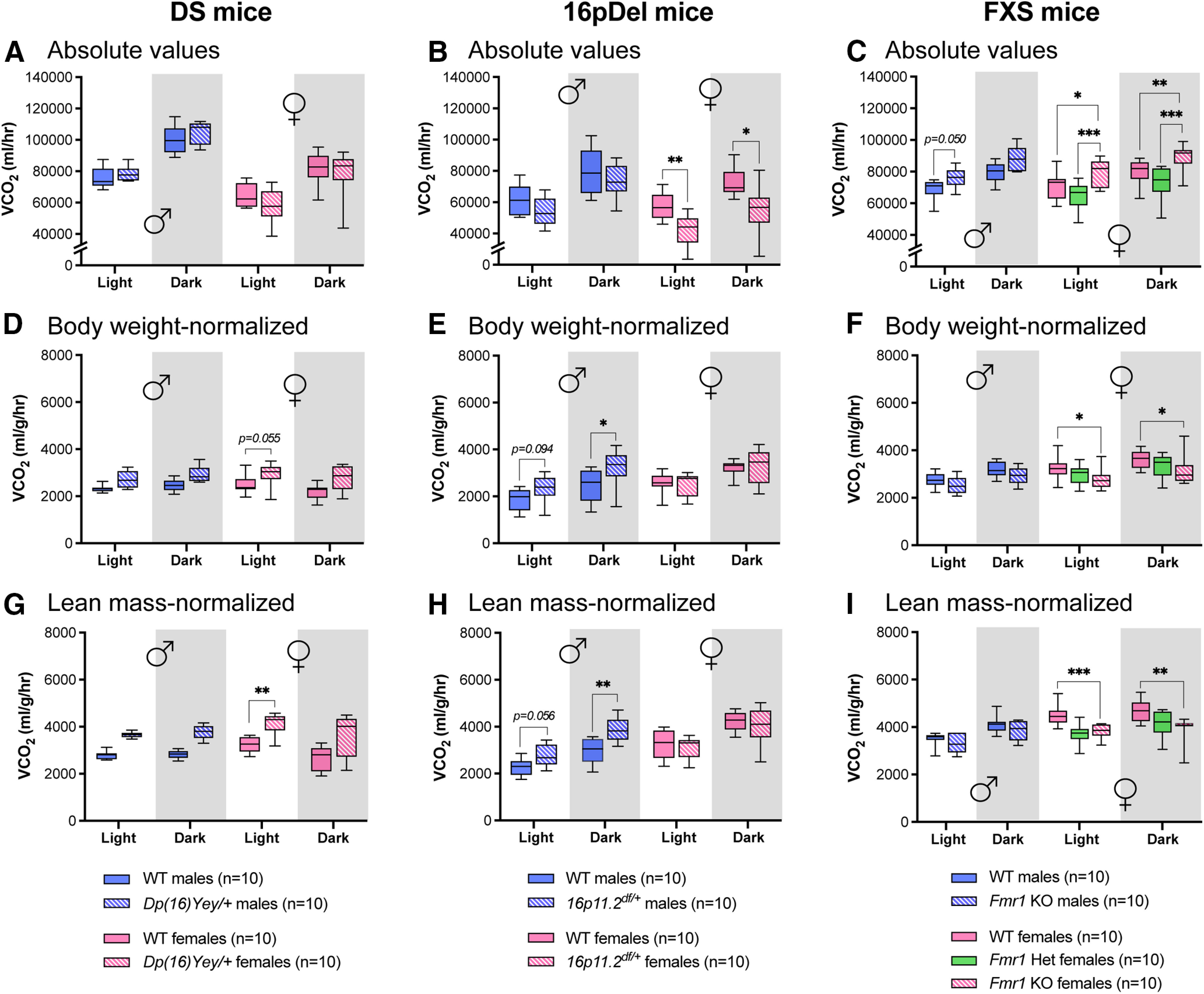
VCO_2_ by DS, 16pDel, and FXS mice measured in CLAMS. ***A–C***, Absolute values of average VCO_2_ during day (light) and night (dark) in WT and *Dp(16)Yey/+* male and female mice (***A***); WT and *16p11.2^df/+^* male and female mice (***B***); WT and *Fmr1* KO (homo/heterozygous) male and female mice (***C***). ***D–F***, Average VCO_2_ during day and night normalized to body weight in WT and *Dp(16)Yey/+* male and female mice (***D***); WT and *16p11.2^df/+^* male and female mice (***E***); WT and *Fmr1* KO (homo/heterozygous) male and female mice (***F***). ***G–I***, Average VCO_2_ during day and night normalized to lean mass in WT and *Dp(16)Yey/+* male and female mice (***G***); WT and *16p11.2^df/+^* male and female mice (***H***); WT and *Fmr1* KO (homo/heterozygous) male and female mice (***I***). The night phase of testing is depicted by gray shaded areas. WT, wild type; DS, Down syndrome; 16pDel, 16p11.2 deletion syndrome; FXS, fragile X syndrome. Data are whisker boxes (min to max, center line indicating median); *n* = 10 animals per group. Asterisks depict significant differences between groups; *p <* *0.05, **p <* *0.01, ***p <* *0.001 (two-way ANOVA and Sidak’s *post hoc* test); *p* value is indicated when approaching significance. ♂: males; ♀: females.

The CLAMS also measure fluctuations in CO_2_ production (VCO_2_), which also reflects changes in resting metabolism (Farinatti et al., 2016; Patel et al., 2020). In absolute values for the DS model, females produced overall slightly more CO_2_ than their male counterparts (Fig. 4*A*). Lean mass-normalized VCO_2_ values revealed that *Dp(16)Yey/+* females produced significantly more CO_2_ than their WT counterparts during the day (Fig. 4*G*). In absolute values, *16p11.2^df/+^* females produced significantly less CO_2_ than their WT littermates (Fig. 4*B*). However, weight-normalized and lean mass-normalized VCO_2_ values appeared higher in *16p11.2^df/+^* males at night compared with WT littermates (Fig. 4*E*,*H*). In absolute values, *Fmr1*^−/−^ females displayed higher VCO_2_ compared with their WT counterparts throughout the 24-h cycle (Fig. 4*C*). However, normalized VCO_2_ values revealed that *Fmr1*^−/−^ females have significantly reduced CO_2_ production compared with WT littermates (Fig. 4*F*,*I*). These data demonstrate that genetic variations associated with DS, 16pDel, and FXS lead to distinct rates of CO_2_ production and that these phenotypes are sex specific.

**Figure 5.**
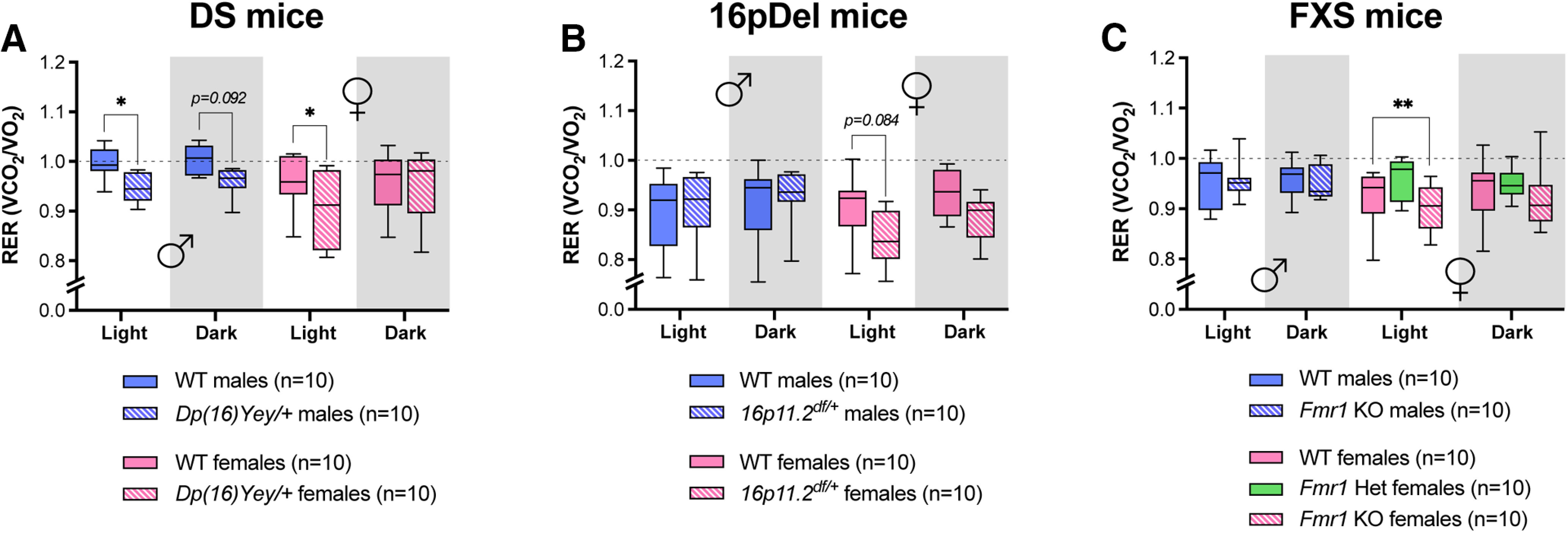
RER from DS, 16pDel, and FXS mice. ***A–C***, RER calculated from absolute values during day (light) and night (dark) in WT and *Dp(16)Yey/+* male and female mice (***A***); WT and *16p11.2^df/+^* male and female mice (***B***); WT and *Fmr1* KO (homo/heterozygous) male and female mice (***C***). The night phase of testing is depicted by gray shaded areas. The dotted line depicts a RER of 1. WT, wild type; DS, Down syndrome; 16pDel, 16p11.2 deletion syndrome; FXS, fragile X syndrome. Data are whisker boxes (min to max, center line indicating median); *n* = 10 animals per group. Asterisks depict significant differences between groups; *p <* *0.05, **p <* *0.01 (two-way ANOVA and Sidak’s *post hoc* test); *p* value is indicated when approaching significance. ♂: males; ♀: females.

Obtaining VO_2_ and VCO_2_ allows for calculation of the respiratory exchange (RER = VCO_2_/ VO_2_), which indicates the preferred metabolic substrate. An RER value near 1 is indicative of predominant usage of carbohydrates as energy substrates (Ramos-Jimenez et al., 2008; Farinatti et al., 2016; Coffman et al., 2019; Patel et al., 2020). All mice housed in the CLAMS for this study displayed an RER of ∼1 and below, consistent with low-to-moderate activity (Fig. 5). During their inactive (light) phase, male and female *Dp(16)Yey/+* mice displayed a significantly lower RER than their WT counterparts (Fig. 5*A*). No significant difference in RER was detected between WT and *16p11.2^df/+^* mice (Fig. 5*B*), whereas *Fmr1*^−/−^ females displayed a lower average RER during the day compared with WT females (Fig. 5*C*). These data reveal distinct sex-specific effects of genetic variations on basal energy metabolism in the DS, 16pDel, and FXS mouse models.

**Figure 6.**
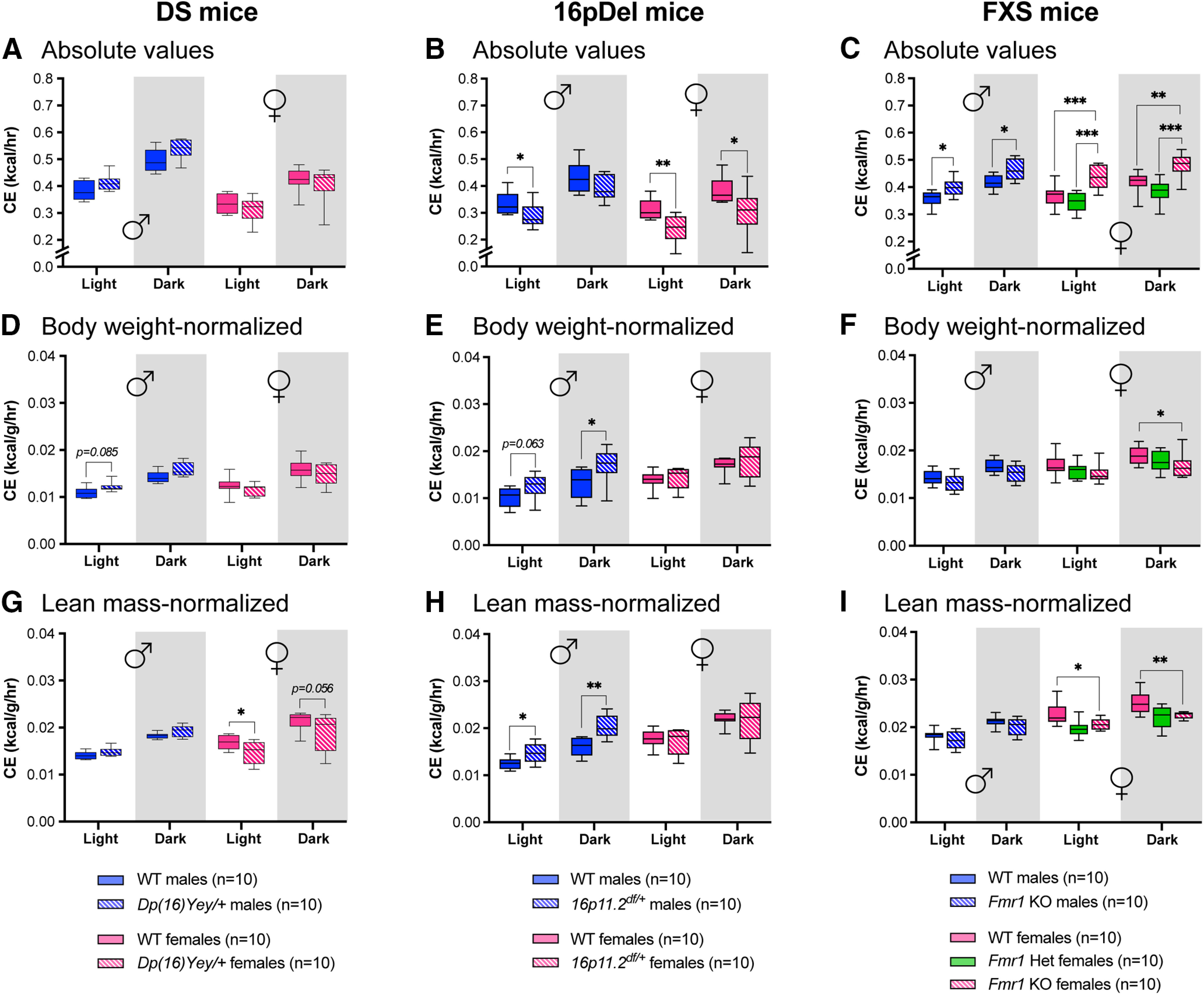
CE by DS, 16pDel, and FXS mice measured in CLAMS. ***A–C***, Absolute values of average CE during day (light) and night (dark) in WT and *Dp(16)Yey/+* male and female mice (***A***); WT and *16p11.2^df/+^* male and female mice (***B***); and WT and *Fmr1* KO (homo/heterozygous) male and female mice (***C***). ***D–F***, Average CE during day and night normalized to body weight in WT and *Dp(16)Yey/+* male and female mice (***D***); WT and *16p11.2^df/+^* male and female mice (***E***); and WT and *Fmr1* KO (homo/heterozygous) male and female mice (***F***). ***G–I***, Average CE during day and night normalized to lean mass in WT and *Dp(16)Yey/+* male and female mice (***G***); WT and *16p11.2^df/+^* male and female mice (***H***); and WT and *Fmr1* KO (homo/heterozygous) male and female mice (***I***). The night phase of testing is depicted by gray shaded areas. WT, wild type; DS, Down syndrome; 16pDel, 16p11.2 deletion syndrome; FXS, fragile X syndrome. Data are whisker boxes (min to max, center line indicating median); *n* = 10 animals per group. Asterisks depict significant differences between groups; *p <* *0.05, **p <* *0.01, ***p <* *0.001 (two-way ANOVA and Sidak’s *post hoc* test). ♂: males; ♀: females.

Finally, the CLAMS monitors heat produced by the animals (in kilocalories per hour) as an indicator of CE. In the DS mouse model, females displayed slightly lower CE compared with males in absolute values (Fig. 6*A*). Interestingly, *Dp(16)Yey/+* females showed significantly reduced CE compared with WT females when values were normalized to lean mass (Fig. 6*G*). In absolute values, *16p11.2^df/+^* mice displayed reduced CE compared with sex-matched WT littermates (Fig. 6*B*). However, when normalized to body weight or lean mass, *16p11.2^df/+^* males displayed increased CE (Fig. 6*E*,*H*). *Fmr1*^−/−^ mice displayed significantly higher absolute CE compared with WT littermates, a phenotype again more pronounced in females (Fig. 6*C*). However, when normalized to body weight or lean mass, *Fmr1*^−/−^ females displayed significantly reduced CE (Fig. 6*F*,*I*). These results demonstrate that the DS, 16pDel, and FXS mouse models display distinct, sex-specific levels of CE.

### Plasma metabolites measured by LC-MS in DS, 16pDel, and FXS mice

**Figure 7.**
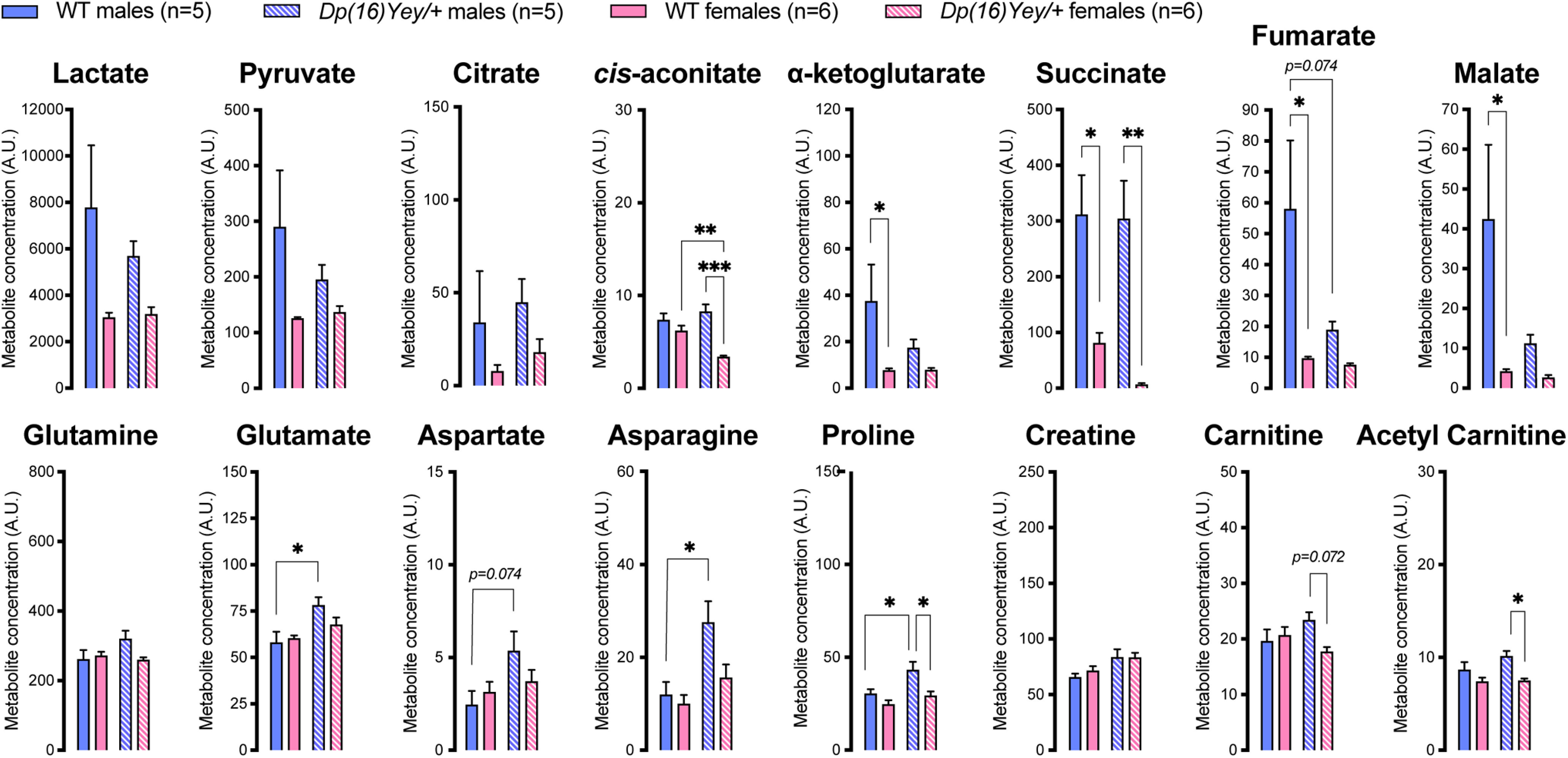
Plasma metabolite levels in DS mice measured by LC-MS. WT, wild type. Data are mean ± SEM; *n* = 5–6 animals per group. Asterisks depict significant differences between groups; *p <* *0.05, **p <* *0.01, ***p <* *0.001 (two-way ANOVA and Tukey’s *post hoc* test).

**Figure 8.**
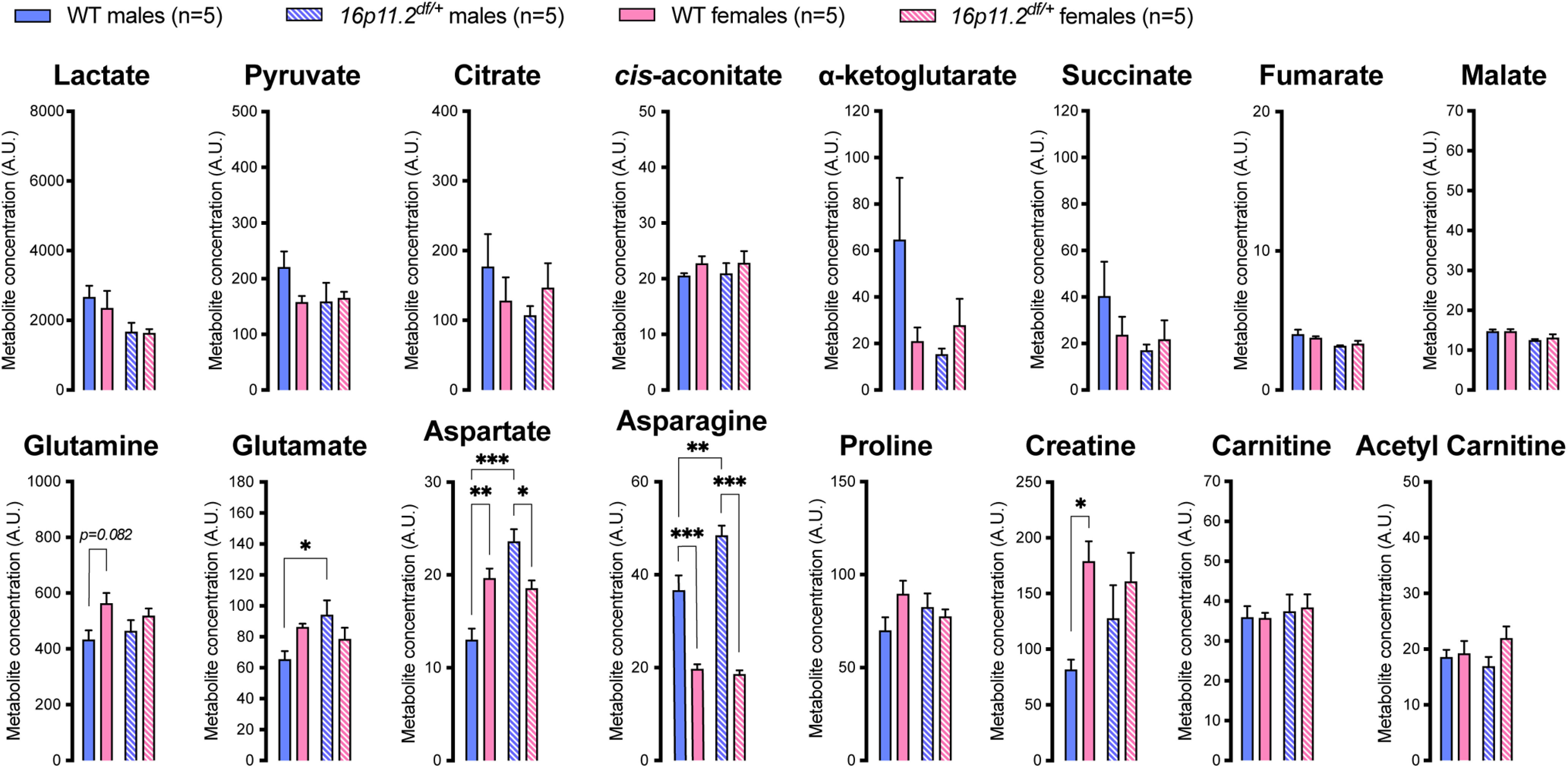
Plasma metabolite levels in 16pDel mice measured by LC-MS. WT, wild type. Data are mean ± SEM; *n* = 5 animals per group. Asterisks depict significant differences between groups; *p <* *0.05, **p <* *0.01, ***p <* *0.001 (two-way ANOVA and Tukey’s *post hoc* test).

**Figure 9.**
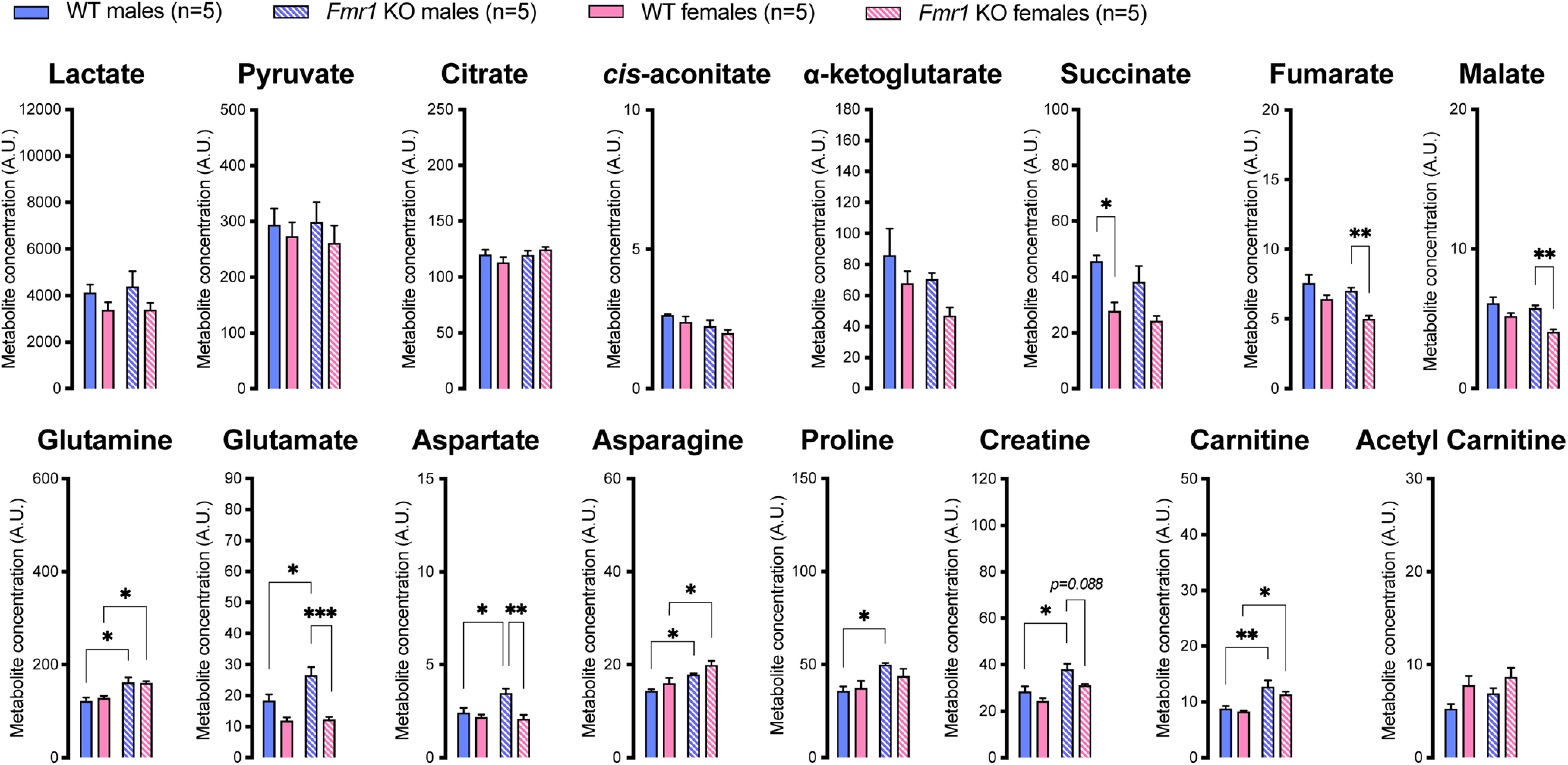
Plasma metabolite levels in FXS mice measured by LC-MS. WT, wild type. Data are mean ± SEM; *n* = 5 animals per group. Asterisks depict significant differences between groups; *p <* *0.05, **p <* *0.01, ***p <* *0.001 (two-way ANOVA and Tukey’s *post hoc* test).

Since the above-mentioned findings suggested sex-specific alterations in basal energy metabolism, and as recent studies have linked NDD to alterations in plasma metabolites related to one-carbon and energy metabolism (Gross et al., 2019; Orozco et al., 2019), we next sought to identify differences in circulating metabolites related to mitochondrial function in all three mouse models. Using targeted metabolomics by LC-MS, we measured the levels of key plasma metabolites related to the tricarboxylic acid (TCA) cycle in three independent batches (DS, 16pDel, and FXS mice; Figs. 7-9). These metabolites include intermediates of the TCA cycle (e.g., citrate, *cis*-aconitate and α-ketoglutarate) as well as anaplerotic metabolites that replenish the TCA cycle intermediates (e.g., pyruvate, aspartate, glutamate). Blood plasma was collected in the fed state to match CLAMS analyses that were performed with free access to food and water.

When compared with their respective sex-matched WT littermates, *Dp(16)Yey/+* females displayed significantly lower levels of *cis*-aconitate, while *Dp(16)Yey/+* males exhibited higher concentration of glutamate, asparagine and proline (Fig. 7). Compared with *Dp(16)Yey/+* females, *Dp(16)Yey/+* males displayed higher levels of *cis*-aconitate, succinate, proline, and acetyl carnitine (Fig. 7).

When compared with their WT counterparts, *16p11.2^df/+^* females did not show any change, while *16p11.2^df/+^* males displayed significantly higher plasma concentrations of glutamate, aspartate and asparagine (Fig. 8). *16p11.2^df/+^* males also displayed higher levels of aspartate and asparagine compared with *16p11.2^df/+^* females (Fig. 8).

Finally, compared with their respective sex-matched WT littermates both *Fmr1*^−/−^ males and females displayed significantly elevated levels of glutamine, asparagine and carnitine (Fig. 9). *Fmr1* KO males also displayed a significant increase in glutamate, aspartate, proline, and carnitine compared with their WT counterparts. *Fmr1*^−/−^ females showed significantly lower levels of fumarate, malate, glutamate, and aspartate than *Fmr1*^−/−^ males (Fig. 9).

## Discussion

Together, our study provides the first systematic characterization of basal metabolism in mouse models of DS, 16pDel, and FXS, revealing that these models display distinct, sex-specific basal metabolic signatures with minimal influence of physical activity and independent of the number of calories consumed.

Activity data obtained in our study do not necessarily reflect the hyperactivity phenotypes commonly found among human or rodent NDDs, a discrepancy most likely because of our experimental setup. Indeed, the CLAMS consists of relatively small individual cages, limiting animal mobility. For instance, in a normal home cage environment, male and female *16p11.2^df/+^*mice are hyperactive (Horev et al., 2011; Ouellette et al., 2020), and the majority of 16p11.2 deletion human carriers display hyperactivity (Miller et al., 2009). However, the limited space offered by CLAMS cages serves to control for metabolic measures, enabling accurate assessment of basal metabolism independent of activity. Of note, whereas hyperactivity is a common feature among individuals with NDDs, they are, however, less likely to engage in physical activity (Vis et al., 2009; Mendonca et al., 2010; Mazurek and Wyka, 2015), partly because of a lack of social skills necessary to participate in group-based physical activities (Foerste et al., 2016).

Results obtained from the DS mouse model suggest that differences in metabolism arise from the DS genotype independent of important factors that influence metabolism, including activity levels and food intake. Pronounced differences were found between mutant (*Dp(16)Yey/+)* males and females, bolstering the importance of sex differences in DS research. Sex differences in human DS include higher disease penetrance among males and high rates of congenital heart defects and increased abdominal adiposity in females (Sobey et al., 2015; Flygare Wallén et al., 2018). Our results also suggest that DS phenotypes are more severe in male mice. The fact that *Dp(16)Yey/+* mice modeling DS do not display differences in weight and lean mass in our study also highlights an important discrepancy with the human syndrome. This could reflect the particular DS mouse model used, since *Dp(16)Yey/+* mice only possess trisomy of ∼66% of Hsa21 gene orthologues (Li et al., 2007; Roubertoux and Carlier, 2010; Yu, 2010; Herault et al., 2017). The other Hsa21 gene orthologues for the mouse are present on Mmu 10 and 17. Although Hsa21 is syntenic with portions of three mouse chromosomes, trisomy of those genes present on Mmu 16 (113 genes) is generally considered a reliable model of human DS cognitive, physical and behavioral phenotypes. The lack of differences in weight and lean mass in the *Dp(16)Yey/+* mice could suggest that the genes linked to human DS-associated obesity are present in regions of synteny on Mmu17 or Mmu10. However, body proportions of a DS mouse model harboring trisomy of all Hsa21 syntenic regions found small but significant reductions in body length and weight (Yu, 2010), again in discordance with human DS body proportions (Vis et al., 2009; Shields et al., 2017).

The *16p11.2^df/+^* mutation also had an influence on metabolism, yet distinct from the other NDD models used in this study. Results in *16p11.2^df/+^* mice were also independent of activity levels and food consumption. Although *16p11.2^df/+^* mice accurately embody numerous phenotypes reminiscent of the human syndrome, interpretation of metabolic data must be made in light of the opposite body proportions often observed between mutant mice and human carriers (Horev et al., 2011; Portmann et al., 2014; Arbogast et al., 2016). Indeed, human 16pDel is associated with obesity, whereas *16p11.2^df/+^* mice display a propensity for leanness (Horev et al., 2011; Portmann et al., 2014; Arbogast et al., 2016), particularly in females as confirmed by our study.

Finally, *Fmr1*^−/−^ mice also displayed a metabolic phenotype distinct from the DS and 16pDel mouse models. The *Fmr1* mutation was associated with an increase in body weight and altered all physiological indices examined in this study. Interestingly, heterozygous *Fmr1*^+/−^ female mice displayed similar metabolic signatures as WT (*Fmr1^+/+^*) females and males, as expected from compensation by the remaining WT allele (Hoogeveen and Oostra, 1997; Linden et al., 1999; McLennan et al., 2011; Bartholomay et al., 2019).

Remarkably, the 16pDel and FXS mouse models displayed largely contrasting phenotypes. The 16pDel and FXS mouse models also exhibited larger changes in metabolic phenotypes among females compared with males. Both the DS and 16pDel mouse models demonstrated metabolic difference among males and females, regardless of genotype. This could be an effect of the background strain of these mouse models, which used a mixed B6/129 background, whereas the FXS mouse model used a mixed FVB/129 background.

In line with sex-specific and model-specific alterations in body composition and metabolic phenotypes, our metabolomic data suggest significant changes in mitochondrial metabolism. First, many changes were observed for TCA intermediates downstream of citrate (e.g., *cis*-aconitate and succinate in DS mice) to suggest that the first TCA reaction, i.e., condensation of oxaloacetate with acetyl-CoA catalyzed by citrate synthase, is not affected. In agreement, circulating levels of pyruvate and acetyl carnitine were mostly similar in males and females whatever the mouse model and mutation. Second, some TCA intermediates were highly different between male and female WT littermates of the *Dp(16)Yey/+* strain (e.g., succinate, fumarate, and malate), while they were unchanged in the plasma of the other WT strains. This suggests that inter-strain differences of the genetic backgrounds could contribute to these observations. Third, important differences were observed in the levels of anaplerotic substrates in males of each mouse model. The mutations led to increased levels of anaplerotic amino acids including glutamate, aspartate and asparagine in the three mouse models as well as increased proline in *Dp(16)Yey/+* and *Fmr1*^−/−^ males. Interestingly, this pattern was not related to differences in body weight or lean mass (a main source of amino acids) since each model showed either unchanged (Fig. 1*A*), decreased (Fig. 1*B*), or increased (Fig. 1*C*) body weight and lean mass. Increased levels of plasma anaplerotic amino acids could result from increased muscular proteolysis, decreased glucogenesis and/or decreased oxidation and anaplerotic influx in the TCA cycle (Chang and Goldberg, 1978; Owen et al., 2002). Interestingly, altered plasma levels of glutamate, aspartate and asparagine have been associated with prediabetes (Owei et al., 2019) as well as coronary heart disease and type 2 diabetes in human subjects (Ottosson et al., 2018). Although it is premature to speculate based on plasma metabolite levels, which metabolic pathway(s) are affected and whether or not mitochondrial anaplerotic flux is altered, our findings highlight major sex-specific and mutation-specific differences in TCA cycle metabolites and anaplerotic amino acids.

Our findings bring forward the concept of NDD-associated metabolic disturbances and support NDD mouse models as valuable tools to gain novel insight into this understudied association. Providing novel insight into NDD-associated metabolic alterations is an essential prerequisite for future preclinical and clinical investigations. Because of similarities in phenotypic expression, NDDs are generally treated as homogeneous groups, neglecting their unique genetic variations. Our results challenge this idea by demonstrating the influence of genetics despite similarities in overt expression of symptoms. This suggests that personalized clinical interventions may be required to address unique NDD-associated metabolic abnormalities depending on both genetic cause and sex.

Insights into basal metabolic function in live animals will provide a basis for future studies aimed at understanding mechanisms underlying metabolic dysfunction in NDDs, and/or aimed at detecting changes in response to intervention.

## Synthesis

Reviewing Editor: Dritan Agalliu, Columbia University Medical Center Decisions are customarily a result of the Reviewing Editor and the peer reviewers coming together and discussing their recommendations until a consensus is reached. When revisions are invited, a fact-based synthesis statement explaining their decision and outlining what is needed to prepare a revision will be listed below. The following reviewer(s) agreed to reveal their identity: Dwight German, Sharmilla Banerjee-Basu.

The reviewers are split in their decisions. However, after consultation they both agree that the manuscript should be revised to include some of the concerns raised by Reviewer # 2.

Reviewer # 1:

This manuscript provides fundamental data for metabolic and sex differences among three different mouse models of neuro-developmental disorders. The manuscript is very well written and the findings very useful for those studying animals of NDDs.

Reviewer # 2:

In this study, the authors attempted to provide metabolic signatures for three different syndromes. However, the experiments reported in this manuscript are inadequate to make such a bold claim.

Major:

1) Experiments reported here are inadequate to establish a unique metabolic signature with confidence. The impact of this study could be substantially improved by performing additional experiments such as cellular assays in peripheral tissues or defining metabolic markers in blood in these models.

Minor:

1) The abstract should include specifics of the unique metabolic signatures that authors are reporting in the paper. The important findings of this study should be highlighted in the abstract.

2) Running title: Altered basal metabolism in autism mouse models - is misleading as the Down Syndrome mouse model is not generally considered as high fidelity autism model.

## Author Response

We would like to sincerely thank both Reviewers for the time spent on carefully evaluating our

manuscript and for their insightful feedback. Specific comments from Reviewer #2 motivated us

to ameliorate data analysis and reporting, as well as to perform new experiments and to profoundly

rework the manuscript for strengthening conclusions. In brief, major changes implemented to the

revised version include:

- New data (targeted plasma metabolite quantifications);

- Improved data analysis/reporting to meet the standards in the field;

- Improved/extended introduction, discussion, methods, and figures for better readability.

We kindly invite the Reviewers to read the new manuscript in full, as it has been significantly rewritten to address all concerns. Here below are our point-by-point responses to Reviewers.

Reviewer comments/critiques appear in black and our responses in blue:

Response to Reviewer # 1:

We would like to thank Reviewer #1 for her/his thorough expert review. We were very pleased

to read that this Reviewer noted: “This manuscript provides fundamental data for metabolic and

sex differences among three different mouse models of neuro-developmental disorders. The

manuscript is very well written and the findings very useful for those studying animals of NDDs.”

Response to Reviewer # 2:

We would like to thank Reviewer #2 for her/his detailed expert review of our manuscript. After

carefully considering all concerns expressed by Reviewer #2, we indeed better capture why “(...)

the experiments reported in this manuscript are inadequate to make such a bold claim.” We are

now glad to provide a strengthened manuscript that addresses all criticisms, as detailed below.

Major:

1) Experiments reported here are inadequate to establish a unique metabolic signature with

confidence. The impact of this study could be substantially improved by performing additional

experiments such as cellular assays in peripheral tissues or defining metabolic markers in blood

in these models.

We recognize that former figures and tables were insufficient to meet standards in the field of

basal metabolism. We have consulted experts and now present improved figures with more

suitable display including additional normalizations (i.e. to fat and lean mass). In addition, for

simplification, we got rid of the numerous ANOVA data tables since all raw data and protocols

will be made available upon request to the corresponding author at the time of publication.

Finally, to better support our main conclusions we have directly followed the advice of “defining

metabolic markers in blood in these models” and performed targeted plasma metabolomics

focusing on TCA cycle-related metabolites. We are glad to report new striking differences

between the three mouse models, including differences driven by sex and/or genotype within

each mouse model. Minor:

1) The abstract should include specifics of the unique metabolic signatures that authors are

reporting in the paper. The important findings of this study should be highlighted in the abstract.

We have improved the abstract by adding more details about the main findings.

2) Running title: Altered basal metabolism in autism mouse models - is misleading as the Down

Syndrome mouse model is not generally considered as high fidelity autism model.

To address this, we have replaced “autism” by “ASD” in the running title.

As outlined in this rebuttal letter, we have followed the roadmap provided by the Reviewers and

feel confident that these revisions significantly strengthen the conclusions of our manuscript. We

hope that Reviewer #2 will now agree that results we report are timely and remain of interest to

eNeuro’s broad readership
